# Application of Cervical Collars – An Analysis of Practical Skills of Professional Emergency Medical Care Providers

**DOI:** 10.1371/journal.pone.0143409

**Published:** 2015-11-20

**Authors:** Michael Kreinest, Sarah Goller, Geraldine Rauch, Christian Frank, Bernhard Gliwitzky, Christoph G. Wölfl, Stefan Matschke, Matthias Münzberg

**Affiliations:** 1 BG Trauma Center Ludwigshafen, Department of Trauma Surgery and Orthopedics, Ludwigshafen, Germany; 2 PHTLS Europe Research Group, Offenbach/Queich, Germany; 3 University of Heidelberg, Institute for Medical Biometry and Informatics, Heidelberg, Germany; 4 Mittelbaden Clinic, Department for Orthopedic and Trauma Surgery, Baden-Baden, Germany; Toronto Western Hospital, CANADA

## Abstract

**Background/Objective:**

The application of a cervical collar is a standard procedure in trauma patients in emergency medicine. It is often observed that cervical collars are applied incorrectly, resulting in reduced immobilization of the cervical spine. The objective of this study was to analyze the practical skills of trained professional rescue personnel concerning the application of cervical collars.

**Material and Methods:**

Within emergency medical conferences, n = 104 voluntary test subjects were asked to apply a cervical collar to a training doll, wherein each step that was performed received an evaluation. Furthermore, personal and occupational data of all study participants were collected using a questionnaire.

**Results:**

The test subjects included professional rescue personnel (80.8%) and emergency physicians (12.5%). The average occupational experience of all study participants in pre-clinical emergency care was 11.1±8.9 years. Most study participants had already attended a certified training on trauma care (61%) and felt "very confident" in handling a cervical collar (84%). 11% applied the cervical collar to the training doll without errors. The most common error consisted of incorrect adjustment of the size of the cervical collar (66%). No association was found between the correct application of the cervical collar and the occupational group of the test subjects (trained rescue personnel vs. emergency physicians) or the participation in certified trauma courses.

**Conclusion:**

Despite pronounced subjective confidence regarding the application of cervical collars, this study allows the conclusion that there are general deficits in practical skills when cervical collars are applied. A critical assessment of the current training contents on the subject of trauma care must, therefore, be demanded.

## Introduction

Depending on the injury mechanism, numerous trauma patients have injuries of the cervical spine. Current literature states the incidence at 12 out of 100,000 residents [1]. Traffic accidents and falls, but also sports accidents are among the most common causes of spinal injuries [2, 3].

The application of a cervical collar has been a part of standard procedures in trauma patients for many years [4]. Immobilization of the cervical spine is also highly recommended in numerous national and international guidelines for patients who have suffered an accident [5].

However, current literature also describes increasing complications after the application of a cervical collar [6–9]. Incorrectly applied cervical collars result in poorer immobilization of the cervical spine [10]. It must, therefore, be demanded that cervical collars have to be applied correctly.

Despite the increasing spread of certified course formats on care of the severely injured, e. g. PHTLS and ATLS, errors in applying cervical collars to trauma patients are frequently observed in clinical everyday work.

The objective of this study was, therefore, to analyze the practical skills of professional emergency medical care providers concerning the application of a cervical collar in a standardized model test.

## Materials and Methods

The current study has been approved by the ethical committee in charge (Ethics committee of the State Medical Association Rhineland-Palatinate, Mainz, Germany) under the reference number 837.371.13 (9056).

In the year 2013, event participants at various German emergency medical continued training events, conferences, and exhibitions (trained rescue personnel and emergency physicians) were asked to complete a questionnaire and then perform a cervical spinal immobilization on a training doll for this study. Participation in the study took place on a voluntary basis.

At the outset, personal and occupational data were anonymously collected for all test subjects using a questionnaire. Aside from age and current occupation of the participants, occupational experience in emergency care was also surveyed. Furthermore, participation in certified continued education events in emergency medicine, as well as the frequency of applying a cervical collar were documented. Lastly, the test subject was asked to assess how confidently they rated themselves in the use of a cervical collar. A 7-point Likert-scale was used for answering the questions on personal assessment regarding frequency of application and how confident the test subjects felt in doing so. The scale used numbers ranging from -3 (not at all or does not apply at all) via 0 to +3 (applies very frequently or completely).

**Fig 1:**
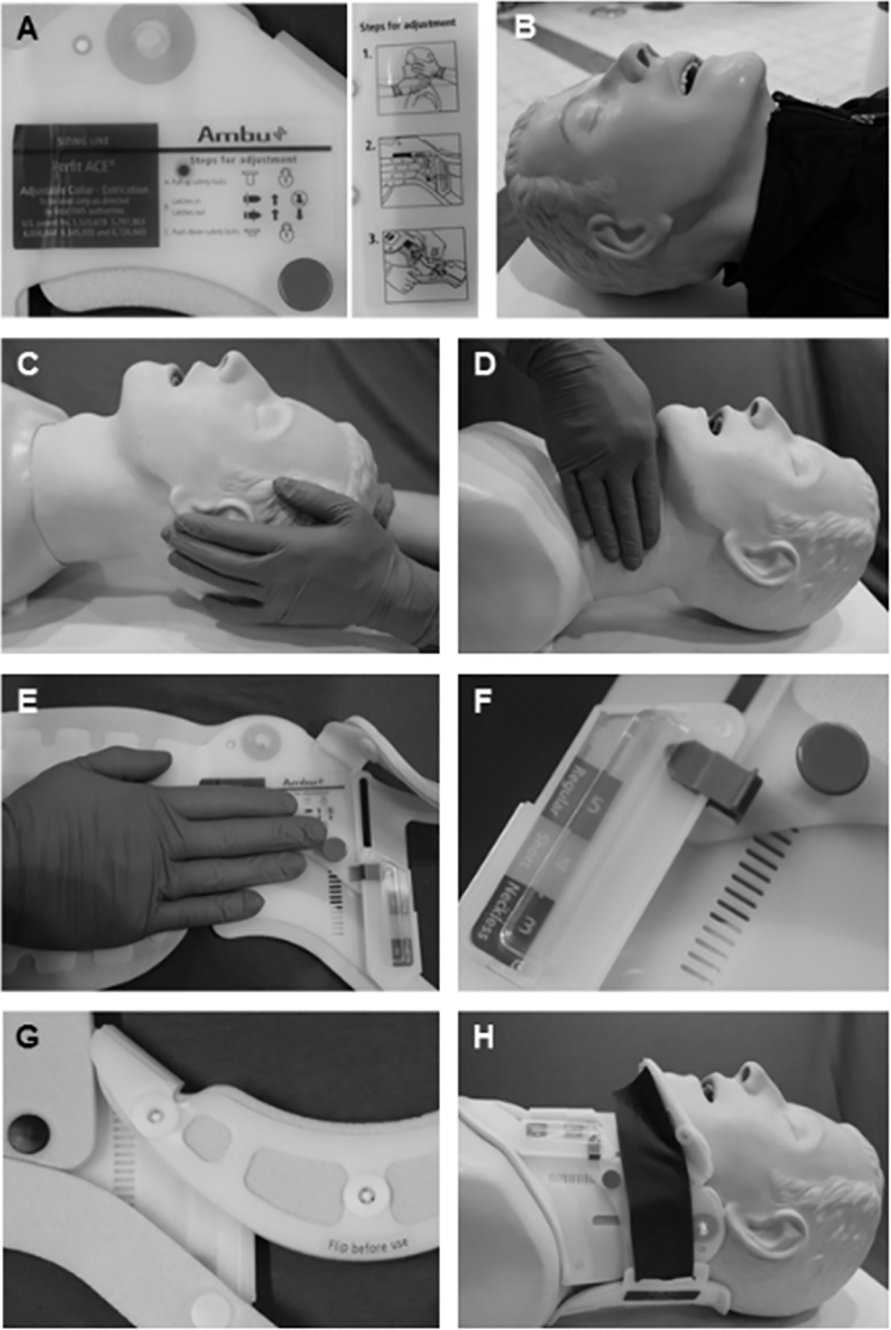
Step by step application of the cervical collar on the model. The utilized cervical collar is supplied by the manufacturer with pictograms on correct use (A). The utilized model (B) should be partially undressed before a helper is instructed to provide manual stabilization of the cervical spine (C). After measuring neck length (D), it is transferred to the cervical collar (E). After setting the required size, the cervical collar is locked (F); the chin section (G) must be folded over to allow correct application of the cervical collar (H).

The test subjects were then asked to apply a commercially available cervical collar (Perfit® ACE^TM^, Ambu, Denmark) to a training doll (Airway Management Trainer, Laerdal, Norway; Fig 1B). The manufacturer of the cervical collar provides brief instructions for use in the form of pictograms on the cervical collar (Fig 1A).

The test subjects were observed by a study supervisor during the application of the cervical collar. The following relevant partial steps were evaluated using a checklist:

- The test subject has to instruct a trained, always available helper (study supervisor) to implement manual stabilization of the cervical spine (Fig 1C).
- Following removal of upper body clothing, the neck length of the model must be determined according to the manufacturer's instructions (Fig 1D).
- The previously determined neck length of the model must be transferred to the cervical collar (Fig 1E). The cervical collar which was used in this study has a "sizing line" for this purpose (Fig 1A). For a perfect fit of the collar to the training doll, the maximum neck length is recommended.
- After setting the previously determined size, the cervical collar must be locked four times (Fig 1F).
- Prior to applying the cervical collar to the training doll, the chin section of the cervical collar must be folded over (Fig 1G).
- Finally, the cervical collar must be correctly applied to the training doll (Fig 1H).

Each of the aforementioned partial steps was evaluated by the study supervisor in a three-level scale (implementation correct, implementation not correct, implementation not provided). Both incorrect implementation and missing implementation were evaluated and documented as "incorrect".

The statistical evaluation of the questionnaire and checklist took place using the program SPSS Statistics 22.0 (IBM, Ehningen, Germany). Categorical variables were evaluated with the statement of absolute and relative frequencies; average parameters and standard deviations were calculated for steady variables. In order to assess associations of completely correct application of the cervical collar and the occupational group, the relative frequencies of correct application of the cervical collar were compared between the applicable occupational groups using the Chi Square test. The association between correct application of a cervical collar and participation in certified continued education were examined in the same manner; likewise the procedure when considering the association between error-free application of the cervical collar and the test subject's subjective sense of confidence.

## Results

N = 104 participants were included into this study. The average age of the participants was 34.5 ± 10.3 years.

The greater share of test subjects consisted of professional rescue personnel (Advanced Life Support-providers) (80.8%) and emergency physicians (12.5%). The remaining test subjects (6.7%) are classified as non-professional assistance personnel in emergency medicine (Basic Life Support-providers). The average occupational experience of all study participants in medical care was 12.8 ± 9.1 years. Average occupational experience of 11.1 ± 8.9 years was stated for pre-clinical emergency care.

Most study participants (61%) stated that they had already attended certified continued education on the theme of trauma care. Out-of-hospital courses has been attended by 41% (PHTLS: 34%; ITLS:- 7%) of the study participants. An ATLS course has been attended by 7% of the study participants. Furthermore, the questionnaire indicated that 84% of the test subjects felt "very confident" in the use of a cervical collar.

The assessment of the application of the cervical collar on the training doll using the checklist showed completely correct implementation of all partial steps in 11% of the tests. Accordingly, incorrect, or absent implementation in at least one partial step was documented in 89% of study participants. The most common errors are shown in Table 1. In particular, the adjustment of the cervical collar size (66%) and locking it (49%) were not correctly implemented.

**Table 1:** Depiction of the most common errors when applying a cervical collar on the model.

Incorrect appearance	Relative frequency	Depiction in Fig 1
Incorrect adjustment of size on the cervical collar	66%	Fig 1 E
Incorrect lock of the cervical collar	49%	Fig 1 F
Incorrect application of the cervical collar on the model	39%	Fig 1 H
Incorrect measurement of size on the model	35%	Fig 1 D

Other partial steps with incorrect implementation included the application of the cervical collar to the model (39%) and the measurement of the neck length of the training doll (35%; Table 1).

No significant difference (Chi-Square-Test: p = 0.407) was shown between the rate of completely correct applications of the cervical collar in the occupational groups of trained rescue personnel as compared to the rate in the group of emergency physicians.

Furthermore, no association was found between the correct implementation of the test and participation in certified continued education on severe trauma care (Chi-Square-Test: p = 0.826). Finally, no correlation was found between completely correct application and the previously stated sense of certainty in handling the cervical collar (Chi-Square-Test: p = 0.862).

## Discussion

The aim of the current study was to examine practical skills in the application of a common commercial cervical collar. To standardize this test, the application of the cervical collar was implemented using a suitable training doll.

In summary, this study showed that only 11% of test subjects were able to apply a cervical collar adequately in all details. Common error sources included the correct size selection on the cervical collar and the correct size measurement on the training doll, among other things. This can lead to the application of an excessively large cervical collar and therefore, increased distraction of the cervical vertebrae. In the presence of an atlanto-occipital dislocation, this can lead to an increased distraction of the cervical spine, which can result in severe complications. Both the application of an excessively large and a too-small cervical collar can result in a significant increase of the range of motion and therefore, reduced immobilization of the cervical spine. It is therefore important that cervical collars are applied appropriately [6, 10].

In this study, however, it was observed that 89% of test implementations resulted in incorrectly applied cervical collars. Incorrect application of a cervical collar occurred both with trained rescue personnel and with emergency physicians. This allows the conclusion that there are general deficits in practical skills when handling cervical collars, in both occupational groups. It is therefore necessary to engage in a critical discussion concerning current training and continued education contents regarding practical skills in applying a cervical collar. The need for more frequent practical training units regarding spinal immobilization was confirmed in a current study [11]. Münzberg et al were able to show that both practical skills and case examples in ATLS courses were evaluated as very helpful by participants [12]. However, this study was able to show that solely attending certified continued education events focusing on severe trauma care (PHTLS, ATLS) is not associated with error-free application of a cervical collar. However, this could be attained at any time by focusing more strongly on practical training in basic methods and techniques, such as immobilization of the cervical spine.

Furthermore, thought should be given to options to improve the sustained effects of such continued education concepts. Short term refresher courses would surely make sense here.

The self-assessments by the study participants in this study (84% of test subjects felt very confident), however, showed a clear discrepancy in relation to the results of the study concerning the technique of applying a cervical collar.

The significance of this study is limited, since not all test subjects were familiar with the provided cervical collar. There are currently numerous different models of cervical collars available whose handling differs significantly in some details [13, 14]. The pictograms, which were provided by the manufacturer on the cervical collar, were only taken into account by few of the participants during the aforementioned test implementation. It remains to be questioned whether this is due to the existing feeling of confidence. Only a few individual participants paid attention to the details of the cervical collar before applying it to the doll. The literature describes models to test cervical collars; according to our knowledge, however, user friendliness is not recorded in this tests [15]. Further studies on applicability and user friendliness of different models of cervical collars should be performed. Additionally, study participants should be given the choice between different models or a general introduction concerning the handling of the current collar should be given by the study’s supervisor in order to reduce bias.

Another limitation of the study consists of the number and selection of included study participants. Participants were asked to participate in the study at emergency medicine conferences, exhibitions, and continued education events. Both the pre-selection of the test subjects by attendance at the corresponding event and the voluntary nature of study participation may result in bias regarding representative random sample selection.

Current literature increasingly shows complications due to the application of a cervical collar. A significant increase in intracerebral pressure and development of brain edema are reported with the application of a cervical collar [7, 8]. Wearing a cervical collar may also significantly reduce the ability to open the patient’s mouth, which can make airway management more difficult. Worsening neurological symptoms of patients with spinal injuries were also reported in individual cases when a cervical collar was applied [9, 16, 17].

Including this literature, the correct application of cervical collars must be urgently recommended, since incorrect applied cervical collars mean that harming the patient cannot be excluded. On the other hand it remains unknown, at what extent of deviation from the most optimal fitting of the cervical collar complications may occur. Further studies should be performed to address this topic.

Despite pronounced subjective confidence regarding the application of cervical collars, trained rescue personnel and emergency physicians show clear deficits in correct implementation. Restructuring of currently existing training and continued education for emergency medical personnel towards focused practical training of basic measures in spinal immobilization must therefore be demanded. Furthermore, every emergency medical care professional must read the instruction manual or get a clear instruction in order to be familiar with the current models of cervical collars used in the own sphere of action.
